# Acridine-Based Antimalarials—From the Very First Synthetic Antimalarial to Recent Developments

**DOI:** 10.3390/molecules26030600

**Published:** 2021-01-24

**Authors:** Mélanie Fonte, Natália Tassi, Paula Gomes, Cátia Teixeira

**Affiliations:** LAQV-REQUIMTE, Departamento de Química e Bioquímica, Faculdade de Ciências, Universidade do Porto, R. do Campo Alegre, 4169-007 Porto, Portugal; melanie.fonte@fc.up.pt (M.F.); natalia.tcpf@gmail.com (N.T.); pgomes@fc.up.pt (P.G.)

**Keywords:** acridine derivatives, antimalarial, hybrid molecules, malaria, *Plasmodium falciparum*, quinacrine

## Abstract

Malaria is among the deadliest infectious diseases in the world caused by *Plasmodium* parasites. Due to the high complexity of the parasite’s life cycle, we partly depend on antimalarial drugs to fight this disease. However, the emergence of resistance, mainly by *Plasmodium falciparum*, has dethroned most of the antimalarials developed to date. Given recent reports of resistance to artemisinin combination therapies, first-line treatment currently recommended by the World Health Organization, in Western Cambodia and across the Greater Mekong sub-region, it seems very likely that artemisinin and its derivatives will follow the same path of other antimalarial drugs. Consequently, novel, safe and efficient antimalarial drugs are urgently needed. One fast and low-cost strategy to accelerate antimalarial development is by recycling classical pharmacophores. Quinacrine, an acridine-based compound and the first clinically tested synthetic antimalarial drug with potent blood schizonticide but serious side effects, has attracted attention due to its broad spectrum of biological activity. In this sense, the present review will focus on efforts made in the last 20 years for the development of more efficient, safer and affordable antimalarial compounds, through recycling the classical quinacrine drug.

## 1. Introduction

A century has elapsed since Laveran described Plasmodium parasites and Ross confirmed that they were transmitted by mosquitoes [1]. Still, malaria remains a leading cause of mortality and morbidity worldwide [2]. Although we have been witnessing the decrease in malaria burden over the last decade, prospects for malaria eradication are now threatened by resistance to artemisinin-based combination therapies, the current first-line antimalarial treatment [3], urging the development of new classes of antimalarials. While a vaccine remains elusive, we depend on chemotherapeutic agents to both treat infections and prevent disease. Most available antiplasmodials target the pathogenic blood stages in humans [4]. However, to eradicate malaria, it is mandatory to develop compounds that block parasite transmission, cure the asymptomatic hepatic infection and clear the latent forms in the liver [5]. Therefore, elimination efforts require identification of new drug classes acting at multiple stages of the parasite life cycle. One strategy to accelerate development of antimalarials is to recycle known drug scaffolds [4].

**Figure 1:**
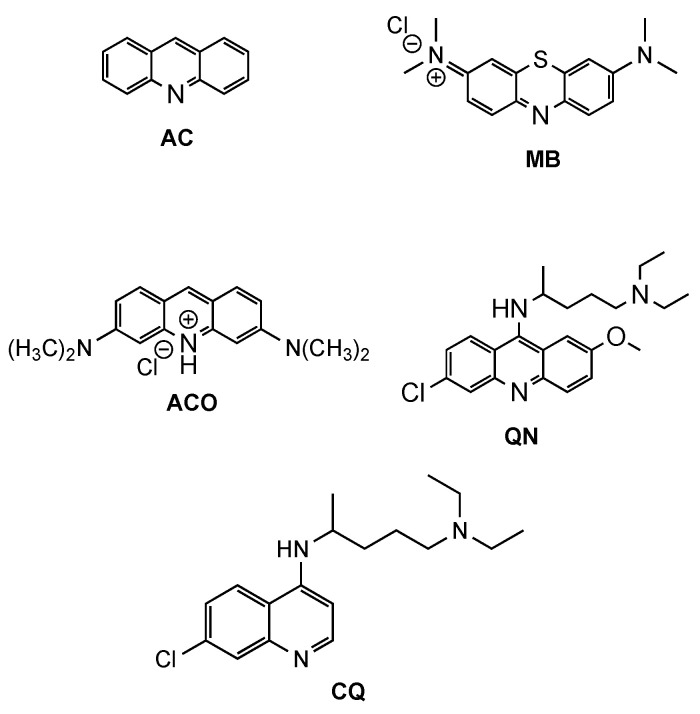
Chemical structure of acridine (**AC**), methylene blue (**MB**), acridine orange (**ACO**), quinacrine (**QN**) and chloroquine (**CQ**).

Acridine (**AC**, Figure 1) derivatives have attracted much attention due to their broad spectrum of biological activity, such as antimicrobial, antitumoral, anti-Alzheimer, antiprionic, antileishmanial and antimalarials agents [4,6,7]. Although different modes of action seem to be exerted by AC derivatives depending on their therapeutic targets, the most consensual mechanism of action (MOA) of AC analogues against different diseases is interaction with DNA [8]. However, other MOAs have been suggested to elucidate the antimalarial activity of AC derivatives, such as inhibition of the parasite’s (i) type II topoisomerases, (ii) mitochondrial bc1 complex, or (iii) hemozoin formation [4]. The interest in AC-based structures as antimalarials occurred from early findings on antiprotozoan/antimicrobial activity of the classic synthetic dyes methylene blue (**MB**, Figure 1) and, later, acridine orange (**ACO**, Figure 1) with IC50 values of 7.8 and 465.7 nM (for **MB** and **ACO**, respectively) against *Plasmodium falciparum* (*Pf*) 3D7 strain [9]. Both the tendency to induce skin coloration and toxicity of **MB** soon triggered the pursuit for more suitable antimalarial substitutes, which led to the synthesis of quinacrine (**QN**, Figure 1), in 1932 [10]. **QN** was thus the first clinically tested synthetic antimalarial drug and was widely used as an antimalarial by soldiers during WWII but was soon superseded by chloroquine (**CQ**, Figure 1), whose bioavailability, safety and efficiency were substantially superior [4,11]. However, with the widespread resistance of *Pf* to **CQ**, the search for more efficient **QN** derivatives or analogues remains vigorous. Amongst different approaches reported in the literature, chemical modifications and the combination or conjugation of AC-based compounds with known relevant pharmacophores have been addressed, in an attempt to outwit resistance mechanisms to classical antimalarials and to produce safer and synergistic or multi-target action compounds. In this context, the present review will focus on efforts made in the last two decades for the development of AC-based compounds as a tool for the rescuing or repurposing of the classical antimalarial quinacrine.

## 2. Quinacrine Derivatives

**Figure 2:**
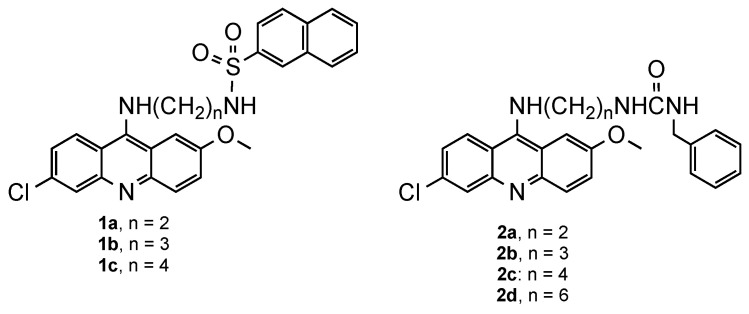
QN derivatives **1** and **2** developed by Chibale and co-workers [12].

Efforts towards the rejuvenation of QN-related compounds started when, in 2001, Chibale et al. [12] developed a series of derivatives in which the QN-acridine core was attached to a second aromatic core via sulphonamide (**1**, Figure 2), or urea linkers (**2**, Figure 2), to improve the solubility of the compounds. Overall, urea derivatives exhibited better activities than sulphonamide derivatives (**1**: 0.010 μg/mL < ED_50_ < 0.033 μg/mL; **2**: 0.0005 μg/mL < ED_50_ < 0.14 μg/mL) against the CQ-sensitive (CQS) *Pf* 3D7 strain, some of them being better than the reference **CQ** (ED_50_ = 0.002 μg/mL). The compound that performed best (**2b**) was further evaluated against the CQ-resistant (CQR) *Pf* K1 strain and presented 4- and 10-fold more potency than **CQ** against CQS and CQR strains (3D7: ED_50_ = 0.0005 μg/mL; K1: ED_50_ = 0.015 μg/mL). However, in addition to the high toxicity in the KB cell line, it was less active against *Pf* K1 than the *Pf* 3D7 strain, suggesting that haem polymerization could be a potential target, such as for 4-aminoquinoline derivatives [12].

**Figure 3:**
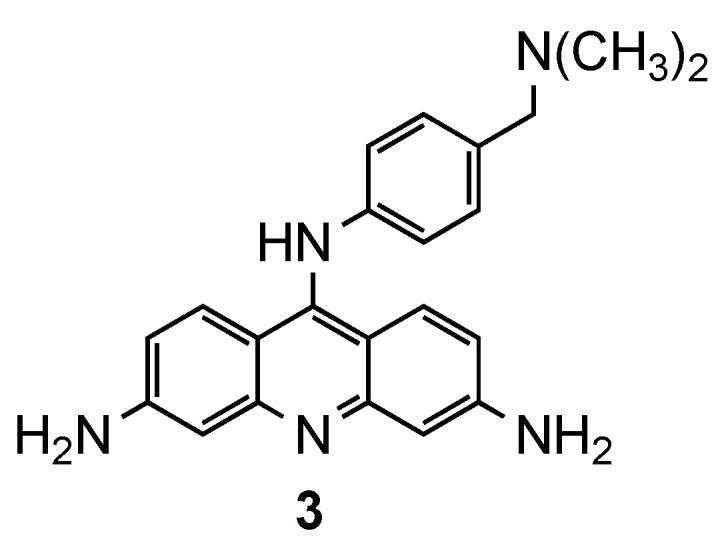
Compound **3** developed by Auparakkitanon and co-workers [13].

Another approach was carried out by Auparakkitanon and co-workers [13], who developed 9-anilinoacridine derivatives targeting two different sites in the malaria parasite, namely, DNA topoisomerase II and hematin formation, to prevent the emergence of resistance. In addition to the evaluation of antimalarial activity against CQS T9/94 and CQR *Pf* K1 strains, the authors also explored the ability of compounds to (i) inhibit in vitro hematin formation, (ii) form drug–hematin complexes and (iii) enhance hematin-induced lysis of red blood cells. Compound **3** (Figure 3) displayed very interesting results, since they (i) exhibited good antimalarial activity, better than the reference **CQ**, by inhibiting DNA topoisomerase II in situ in both strains (K1: IC_50_ (**3**) = 0.034 μM, IC_50_ (**CQ**) = 0.10 μM; T9/94: IC_50_ (**3**) = 0.024 μM, IC_50_ (**CQ**) = 0.59 μM), and (ii) inhibited hematin formation (IC_50_ = 0.125 μM) with an IC_50_ value equivalent to that of **CQ** [13].

**Figure 4:**
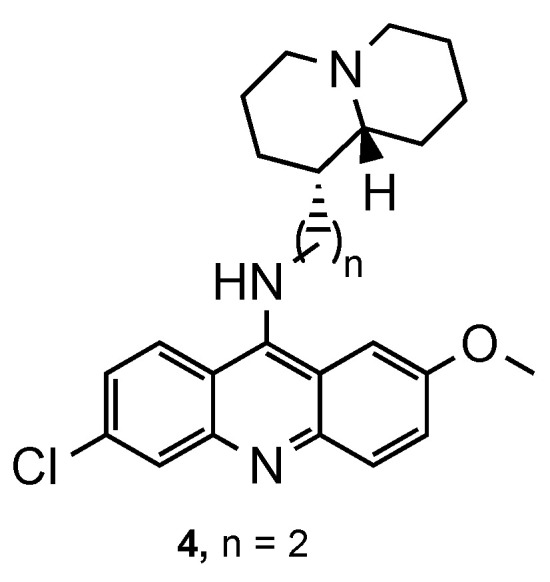
QN derivative **4** developed by Sparatore and co-workers [14].

In an attempt to improve the pharmacokinetic profile of acridine-derivative compounds, Sparatore et al. reported the synthesis of **QN** analogues by linking quinolizidinylalkyl moieties to the 9-amino-6-chloro-2-methoxyacridine core [14]. The activity of the compounds was comparable with **CQ** against the CQS *Pf* D10 strain and three- to four-fold superior in the CQR *Pf* W2 strain. Furthermore, the spacer’s length with 1 and 2 carbons provided more potency compared to 3 carbons. Compound **4** (Figure 4) exhibited the best values of antimalarial activity against both strains (D10: IC_50_ = 30.8 nM; W2: IC_50_ = 68.1 nM) and equimolar cytotoxicity to **QN** on murine cells WEHI. Additionally, the ratio between IC_50_ values of **4** against CQR *Pf* W2 and CQS *Pf* D10 strains suggests the compound’s susceptibility to resistance mechanisms [14].

**Figure 5:**
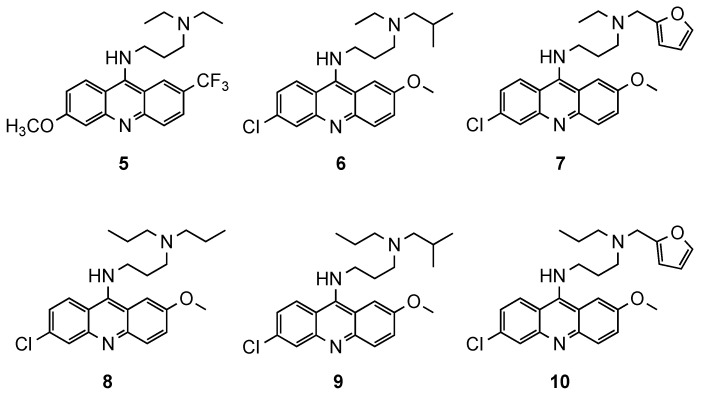
Best compounds (**5**–**10**) developed by Anderson and co-workers [15].

A more conservative approach was performed by Anderson et al., who described the parallel synthesis of 9-aminoacridine analogues by simple changes in **QN**’s side chain [15]. Two libraries (proof-of-concept and cross-coupling) were designed, totaling 175 compounds. Only 93 of the final products had purities suitable for in vitro antimalarial activity evaluation against *Pf* 3D7 and W2 strains. Six compounds (**5**–**10**, Figure 5) demonstrated high in vitro activity against both strains (3D7: 1.0 nM < IC_50_ < 4.1 nM; W2: 1.0 nM < IC_50_ < 7.6 nM), better than the references **QN** (3D7: IC_50_ = 8.1 nM; W2: IC_50_ = 32.1 nM) and **CQ** (3D7: IC_50_ = 7.0 nM; W2: IC_50_ = 382.2 nM). From this set, compounds **5** and **8** stand out due to their sub-nanomolar activity against the *Pf* 3D7 strain and marked improvement of activity against the *Pf* W2 strain (IC_50_ < 1.0 nM). Although these compounds present a high structural similarity to **QN**, activity results suggested some QN side-chain flexibility, as both furyl and alkyl groups were well tolerated [15].

**Figure 6:**
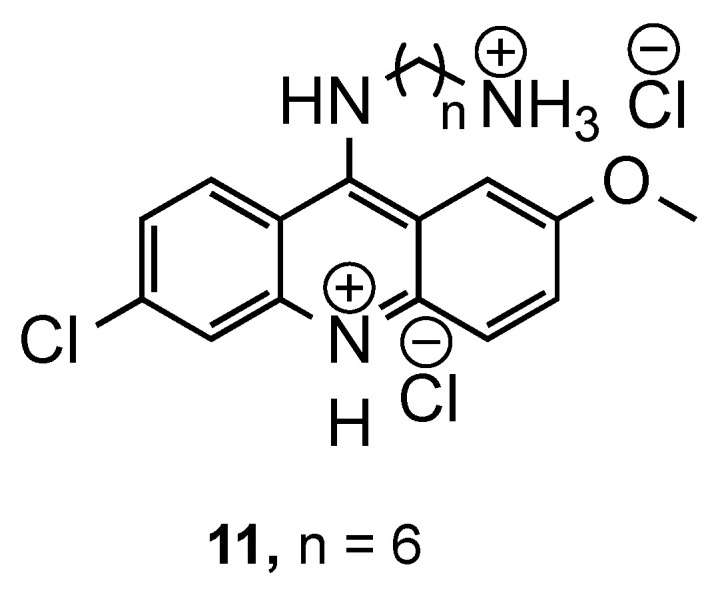
Most active compound **11** developed by Guetzoyan and co-workers [16].

Lucie Guetzoyan et al. [16] explored the importance of a positive charge in the antimalarial activity in two new series of 9-substituted acridyl derivatives. In the first series, the synthesized compounds have several cationic charges: one on the acridine ring and the others on the chains grafted at the 9-amino position. The second series contains only one on the peptidic side chain. Both series were evaluated against one CQS *Pf* strain (3D7) and three CQR *Pf* strains (W2, Bre, and FCR3). Considering the results obtained, the authors concluded that for a good antimalarial activity, the following are necessary: (i) a cationic charge in the acridine core, and (ii) a short chain attached to the acridine ring. Compound **11** (Figure 6) exhibited the best antimalarial activities for all *Pf* strains (0.13 μM < IC_50_ < 0.20 μM), namely, for the CQR strains presenting better IC_50_ values than **CQ** (0.44 μM < IC_50_ < 0.52 μM) [16].

**Figure 7:**
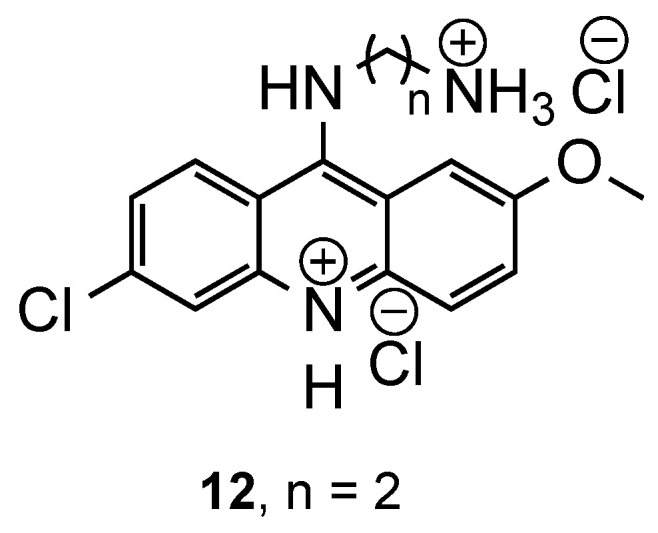
Most active compound **12** developed by Guetzoyan and co-workers [17].

Based on these results, years later, the same authors [17] developed a new series of molecules structurally similar to the previous ones, changing the size and nature of the chain linked to the acridine ring. These modifications were designed to confirm the influence of the (i) chain size, (ii) protonation state of the terminal amino group and (iii) position of methoxy and chlorine substituents in position 2 and 6, respectively (Figure 7). The compounds were evaluated against the same *Pf* CQS and CQR strains as the previous study (3D7, and W2, Bre1 and FCR3, respectively). The results showed that an optimal antimalarial activity requires (i) the presence of the chlorine and methoxy substituents on the acridine ring, and (ii) two positive charges at physiological conditions, one on the acridine ring and one on the terminal function of the side chain. Effectively, compound **12** (Figure 7), which possesses these features, displayed the best results, inhibiting the growth of *Pf* in CQR strains with IC_50_ ≤ 0.33 μM, lower than reference **CQ** (IC_50_ ≤ 0.52 μM). Against the CQS *Pf* 3D7 strain, the compound was slightly less potent (IC_50_ ≤ 0.07 μM) comparatively to the reference (IC_50_ = 0.02 μM) and presented higher toxicity towards KB cells [17].

**Figure 8:**
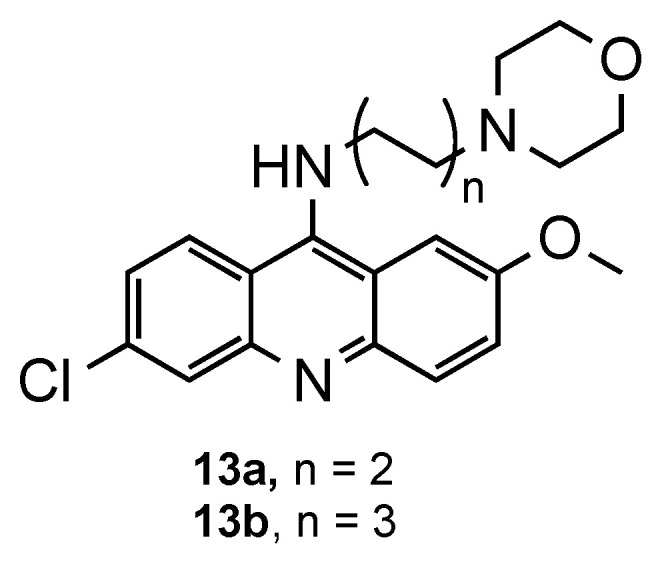
Most promising compound **13** developed by Yu and co-workers [18].

Later, Yu et al. extended the study by introducing terminal heterocyclic groups (piperazinyl, pyrrolidinyl, imidazolyl and morpholinyl) in the aliphatic side chain, since these groups are usually associated with increased bioavailability, metabolic stability and tolerance in humans [18]. As a result, most of the compounds displayed a positive charge at physiological pH, a structural requisite to maintain a good antimalarial activity [16,17]. Morpholinyl derivatives (**13a**–**b**, Figure 8) exhibited better results against CQR (IC_50_ = 30 nM; SI = 3.7–7.2) and CQS (IC_50_ = 9–10 nM; SI = 12.2–21.5) *Pf* strains compared to the references **CQ** and **QN**, alongside with moderate cytotoxicity on KB and MRC-5 cells. These results may be related with their moderate inhibitory activity in β-hematin formation and potent inhibitory activity in topoisomerase VI-mediated DNA relaxation [18].

**Figure 9:**
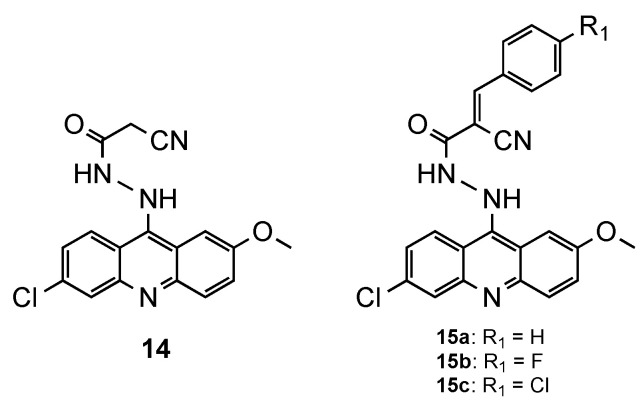
Compounds **14** and **15** developed by Silva and co-workers [19].

Silva et al. [19] synthesized a set of four **QN** derivatives (**14**, and **15**, Figure 9), evaluated their antimalarial activity and correlated those values to a possible interaction with human serum albumin (HSA). The drugs’ interaction with serum proteins provided notable information concerning the pharmacokinetics and pharmacodynamics features, taking into account that only the free drug exerted the pharmacological effect [20]. Furthermore, the interaction with DNA was also evaluated to elucidate the mechanism of action. The four compounds synthesized were active against the CQR *Pf* W2 strain and were less cytotoxic than the references primaquine **(PQ**) and amsacrine (**AMS**) against HepG2 cells. Compound **15a** (Figure 9), which contains a benzylidene group unsubstituted, showed better activity than **PQ**, and similar to AMS against the *Pf* W2 strain (**15a**: IC_50_ = 0.90 μM; **PQ**: IC_50_ = 1.70 μM; **AMS**: IC_50_ = 0.80 μM). Regarding the interaction with HSA, the most active compound presented the lowest interaction with the protein, while the less active derivative showed the highest affinity. Moreover, a high linear correlation was observed between the IC_50_ values of the antimalarial assays and the binding constants in study of interaction with DNA, which suggested that DNA may be the main biological target of these molecules [19].

**Figure 10:**
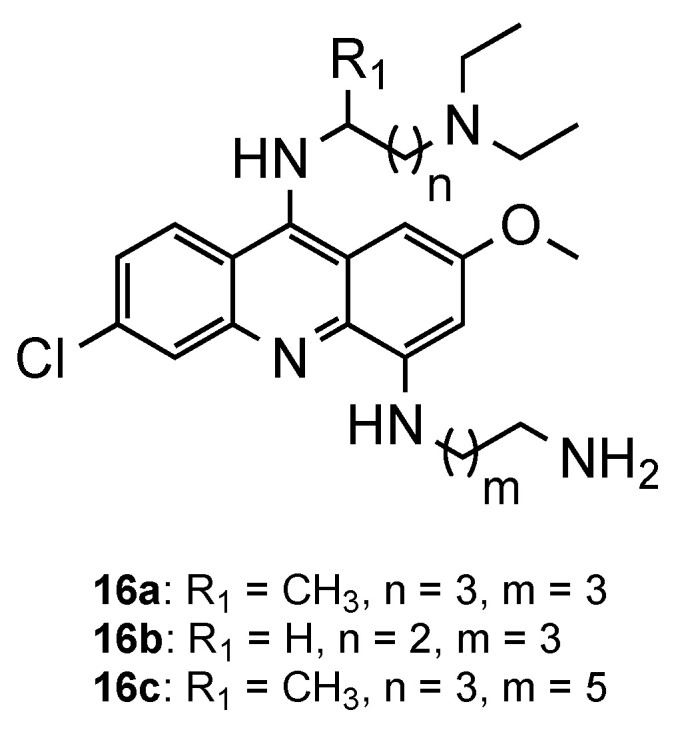
QN derivatives developed by Fonte and co-workers [21].

Recently, Fonte et al. developed **QN** derivatives through the introduction of a like **PQ**-side chain in position 4 of the acridine ring (Figure 10) towards multi-stage antimalarial drugs [21]. After the establishment of the synthetic route of these derivatives [22], the series of three compounds, with different side-chain length, were evaluated for their antimalarial activity against CQS 3D7 and CQR *Pf* W1 strains, and the hepatic stages of *P. berghei*. Overall, all the compounds exhibited activity against both stages of the parasite life cycle. Due to the scarce number of compounds, it was not possible to assess a solid structure–activity relationship. However, the results confirmed the importance of the aminoalkyl chain in position 9 of the acridine ring in the blood antimalarial activity (**16a**–**c**, 3D7: 0.26 µM < IC_50_ < 0.68 µM; W2: 0.49 µM < IC_50_ < 6.17 µM), when compared to the corresponding analogues lacking this side chain (3D7: IC_50_ > 5.64 µM; W2: 2.39 µM < IC_50_ < 5.14 µM). Regarding liver-stage activity, compound **16a** was significantly active at 10 μM (IC_50_ = 11.02 µM), although the other derivatives were highly cytotoxic towards Huh-7 cells at this concentration. Remarkably, none of the compounds was hemolytic up to 10 µM [21].

## 3. Hybrids Containing the 9-Aminoacridine Scaffold

**Scheme 1:**
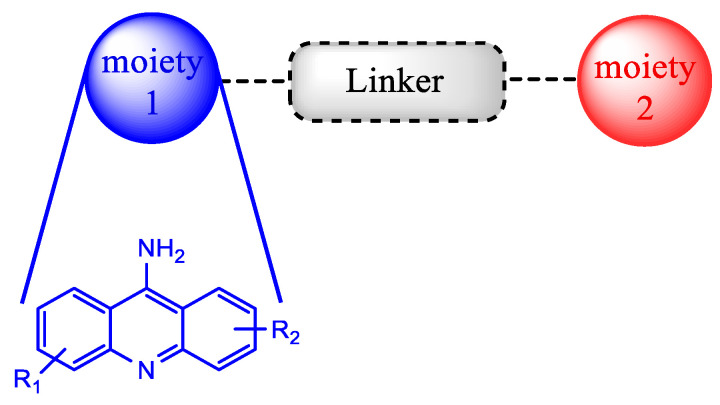
Representative scheme of a **QN** hybrid drug.

Another noteworthy approach used for recycling the **QN** scaffold and obtaining faster and available new antimalarial treatments is based on the concept of hybrid drugs. A hybrid molecule is formed through the covalent link between two known chemical moieties with different biological modes of action. This methodology was developed aiming to obtain molecules with better biologic activity, solubility profile and stability than the parent drugs, as well as less susceptibility to the development of drug resistance (Scheme 1). In the last decade, this strategy has attracted attention, mainly in the field of the development of antimalarial drugs due to the urgency of novel compounds to fight malaria disease [23,24].

### 3.1. Bisacridine Hybrids

**Figure 11:**
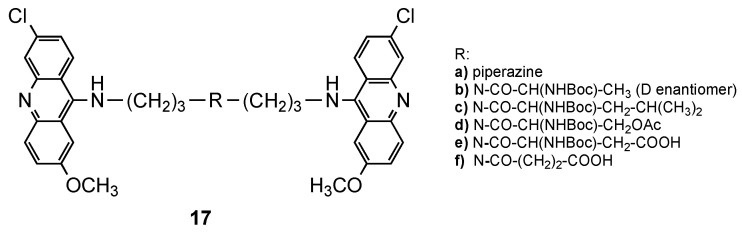
The best compounds **17** developed by Girault and co-workers [26].

Bisacridines were among the first classes of hybrids extensively explored as DNA intercalators. These hybrids are composed of two acridine portions, and studies suggested that the length and flexibility of the spacer between these two acridines affect the intercalation property. The first findings related to this class of compounds came from the work of King et al. [25]. Using spectrophotometric and viscosimetric experiments, they found that a longer and more flexible spacer favored *bis*-intercalation, while bisacridines with a shorter and less flexible spacer monointercalated [25]. The potential of bisacridines as antimalarials was first reported by Girault et al. [26], who developed three series of bisacridines by linking two acridine cores through three different spacers: alkanediamines, linear polyamines or branched polyamines. The in vitro antimalarial activity of all the compounds against a CQR *Pf* FcB1R strain, as well their cytotoxic effects on MRC-5 cells, were evaluated. The best ones (Figure 11) were further evaluated for their efficiency to inhibit the growth of another seven Pf strains (3D7, F32a, GP1, FCR3, FCM29, W2 and K1). Compound **17a** (Figure 11), a piperazine derivative, showed the best results with high and selective activity against different *Pf* strains (IC_50_ values ranging from 8 to 18 nM), and a total absence of cytotoxic effects upon MRC-5 cells and murine macrophages [26].

**Figure 12:**
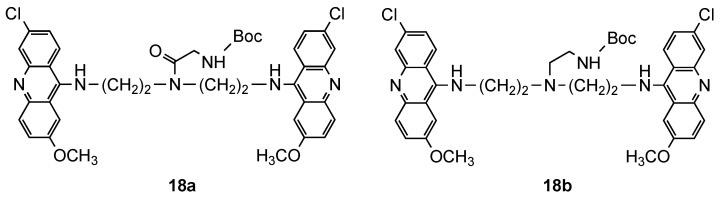
Compounds **18** developed by Girault and co-workers [27].

From their previous work [26], they selected a set of compounds based on the cytotoxicity/activity ratio (**17a**–**f**) and tested their activity upon mice infected by *P. berghei* at the concentration of 40 mg/kg [27]. Two additional compounds (**18a** and **18b**, Figure 12) were synthetized to verify the influence of the bond between the side chain and the polyamine linker on antimalarial activity and cytotoxicity. The lack of solubility of compound **17a** prevent it from being assessed in vivo; however, important considerations were obtained for the other compounds: (i) the most active compounds in vitro [26] (**17a**–**f**) were found to be inactive at the tested concentration; (ii) the *N*-alkylation of the central amino group (**18b**), instead of *N*-acylated (**18a**), generated toxicity in vivo; (iii) the compound **18a** was the most active compound in vivo, with 22% of prolongation of the mean survival time. These results indicated the difficulty to predict relationships between in vitro and in vivo activity and toxicity, even between members of the same family of compounds [27].

Another library of bisacridines was developed by Caffrey et al. [28]. The authors synthesized 16 bisacridines holding different linkers (polyamine, alkylated polyamine, acylated polyamine, alkyl or polyether) and two *bis*-aza-acridines with a polyamine and alkyl linker. The compounds were evaluated for their in vitro antimalarial activity against the CQS *Pf* 3D7 and the CQR *Pf* W2 strains, and their cytotoxicity on HL-60 mammalian cell lines. Structure–activity studies demonstrated some interesting aspects: (i) an increase higher than the minimum linker length (10 Å in this case) increases the activity against the parasite, but also increases the cytotoxicity; on the other hand, (ii) the addition of steric bulk and conformational constraint in the polyamine linker through alkylation, acylation or substitution of polyamine-reduced cytotoxicity retains antimalarial activity; (iii) the polyether linker improves potency relative to the equivalent polyamine linker as well as the substitution of the acridine heterocycle by the aza-acridine.

**Figure 13:**
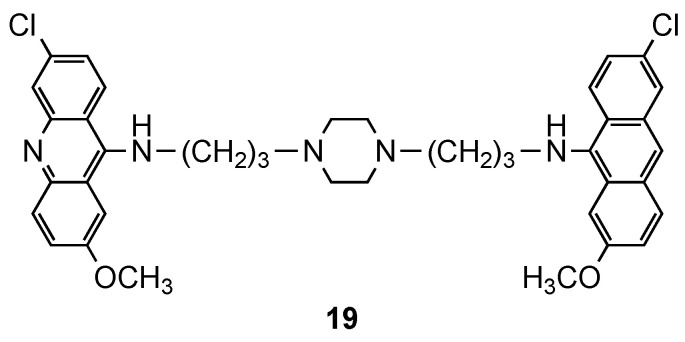
Compound **19** developed by Caffrey and co-workers [28].

Although most of the compounds presented EC_50_ values comparable to references **CQ** and **QN** [28], compound **19** (Figure 13), a piperazinyl derivative, displayed the best therapeutic indices (3D7: EC_50_ = 64 nM; W2: EC_50_ = 112 nM), which supported the results of Girault and colleagues [26,27].

### 3.2. Acridine–Artemisinin Hybrids

**Figure 14:**
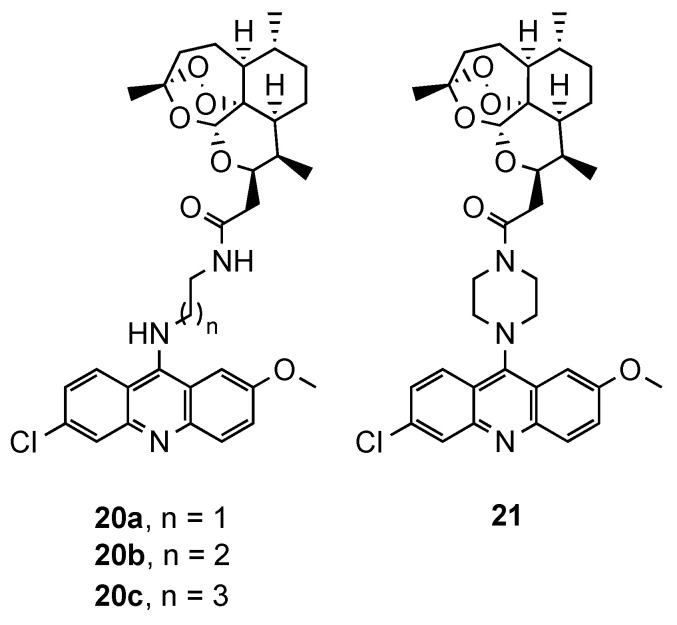
Compounds (**20** and **21**) developed by Jones and co-workers [33].

The relevance of artemisinin-based antimalarials in the current clinical strategies against CQR malaria, and the recent emergence of resistance to ACT [3], also led to the exploration of several hybrid molecules containing artemisinin moiety. The exact mechanism of this first line antimalarial and its derivatives is not completely understood, but it is believed to rely on the cleavage of the endoperoxide bond by reduced Fe^2+^, leading to cytotoxic carbon-centered free radicals and the alkylation of critical biomolecules of the parasite [29,30,31]. In addition to the intrinsic antimalarial activity of the acridine derivatives, their conjugation with artemisinin scaffold is expected to increase the accumulation of the hybrid in the food vacuole, and consequently, the efficiency of artemisinin [32]. One of the first works reporting such hybrids was carried out by Jones et al. [33]. In this study, 1,2,4-trioxane, derived from artemisinin, was covalently linked to 9-diaminoalkyl-6-chloro-2-methoxyacridine moieties through two different spacers (**20**, and **21**, Figure 14). The compounds were evaluated against a CQS *Pf* strain (3D7) and presented IC_50_ values ranging from 5.96 to 289.52 nM. However, even the best compound (**20a**, Figure 14) was less potent than the controls (IC_50_(**20a**) = 5.96 nM; IC_50_(dihydroartemisinin) = 2.30 nM; IC_50_(artemether) = 3.53 nM) [33].

**Figure 15:**
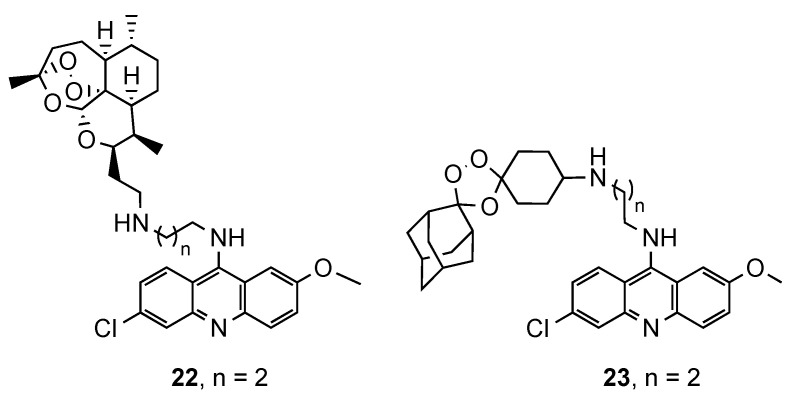
Synthetic and semi-synthetic artemisinin derivatives **22** and **23** developed by Araújo and co-workers [32].

In a similar study, Araújo et al. [32] described the synthesis of a novel series of semi-synthetic trioxaquines and synthetic trioxolaquines, both covalently linked to the 9-diaminoalkyl-6-chloro-2-methoxyacridine moiety (**22** and **23**, Figure 15). The in vitro antimalarial activity, against CQS and CQR *Pf* strains, was evaluated, and both series of compounds were active in a low nanomolar range. In general, the synthetic series were more potent than the semi-synthetic artemisinin derivatives. Compound **22a** (Figure 15) displayed the best results in the semi-synthetic series (IC_50_(3D7) = 12.52 nM; IC_50_(K1) = 14.34 nM), while compound **23a** (Figure 15) was the best in synthetic series (IC_50_(3D7) = 9.67 nM; IC_50_(K1) = 7.20 nM). Both compounds have -(CH_2_)_2_^−^ as a linker, which shows that the activity is very dependent on the nature and length of the linker [32].

**Figure 16:**
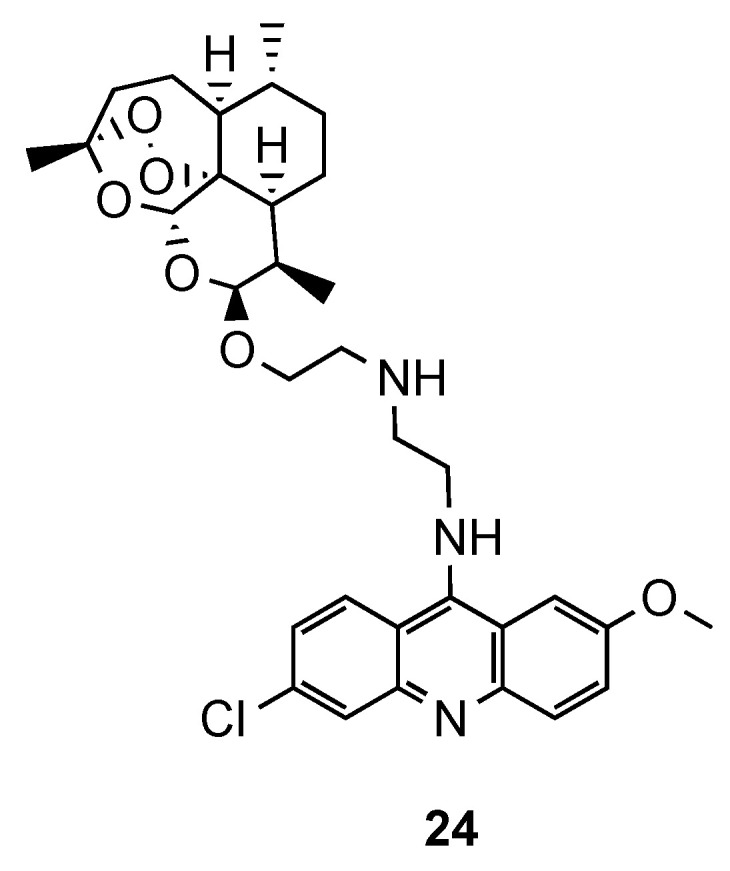
Most active compound **24** developed by Joubert and co-workers [34].

In the same line of thought, Joubert et al. reported the synthesis and in vitro antimalarial activity of hybrids obtained by covalently linking artemisinin and acridine pharmacophores through an aminoethyl ether linker [34]. Most of the compounds were shown to be more active than the reference **CQ**, but less than the references dihydroartemisinin (DHA) and artesunate against CQS and CQR *Pf* strains. Hybrid **24** (Figure 16), which contains ehtylenediamine as a linker, was the most promising antimalarial compound with 7-fold higher antimalarial potency than **CQ** against both strains, along with a high selectivity index towards the parasitic cells (**24**: IC_50_(NF54) = 2.6 nM; IC_50_(Dd2) = 35.3 nM; **CQ**: IC_50_(NF54) = 18.5 nM; IC_50_(Dd2) = 271.7 nM; SI = 615). It is noteworthy that the hybrids possessed generally higher activity than their precursor 9-aminoacridines and less cytotoxic for human cells than DHA [34].

### 3.3. Acridine–Aminoquinoline Hybrids

**Figure 17:**
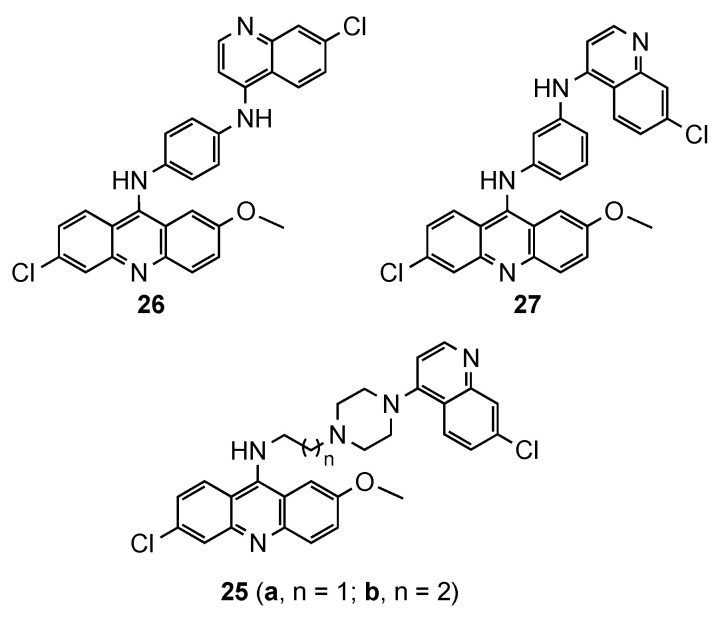
Quinoline–acridine hybrids **25–27** synthesized by Kumar and co-workers [38].

Despite the emergence of resistance to 4-aminoquinoline drugs, this pharmacophore has a crucial role inhibiting the heme polymerization and parasite growing. It is believed that the steric hindrance in bulky compounds, such as bisquinolines, would avoid the drug efflux and overcome the resistance [35,36,37]. However, hybrids containing the acridine and quinoline moieties are scarcely reported. This prominent approach was described by Kumar et al. [38], who synthesized quinoline–acridine hybrids covalently linked by (i) an alkyldiamine chain functionalized piperazine (**25a** and **25b**, Figure 17), or (ii) a *p/m*-phenylenediamine (**26** and **27**, Figure 17). Compound **27** exhibited the best in vitro antimalarial activity against the *Pf* NF54 strain (MIC = 0.25 μg/mL), but still less potent than the reference **CQ** (MIC = 0.125 μg/mL). This compound was tested in vivo in Swiss mice infected with the CQR N-67 strain of *Plasmodium yoelii*, and the results displayed the complete clearance of parasitemia on day 4 at the dose of 50 mg/kg × 4 days by intraperitonial route. However, none of mice survived beyond day 28 [38].

### 3.4. Acridine–Chalcone Hybrids

**Figure 18:**
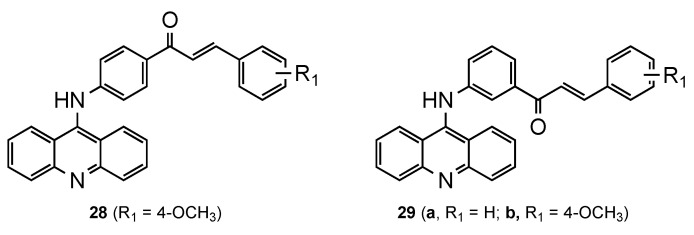
Compounds **28** and **29** developed by Tomar and co-workers [41].

Based on reports of the diverse range of pharmacological activities of chalcones, namely, their ability to defeat plasmodium parasites [39,40], Tomar et al. [41] developed a series of new 9-acridinylamino chalcone derivatives (**28**–**29**, Figure 18). The analogues presented different substituents (-NO_2_, -NH_2_, -OH, -CH_3_, -OCH_3_, -Cl) on chalcone’s ring B at different positions. To complement the structure–activity relationship study, chalcone’s ring A was linked to the 9-aminoacridine in positions 3 or 4. All the compounds were screened for in vitro antimalarial activity against the CQS *Pf* NF54 strain, and it was evident that the location and nature of the substituent(s) on chalcone’s ring B derivatives are essential. The eleven compounds fully inhibited the maturation of parasite at a concentration greater than or equal to 10 μg/mL, namely, compounds **28** and **29a**–**b** exhibited a percentage inhibition above 70% at 2 μg/mL concentration. These three acridine–chalcone hybrids were further screened for in vivo efficacy against a CQR rodent malaria parasite *Plasmodium yoelii* (strain N-67) in a Swiss mice model. However, no significant inhibition in parasitemia was observed [41].

**Figure 19:**
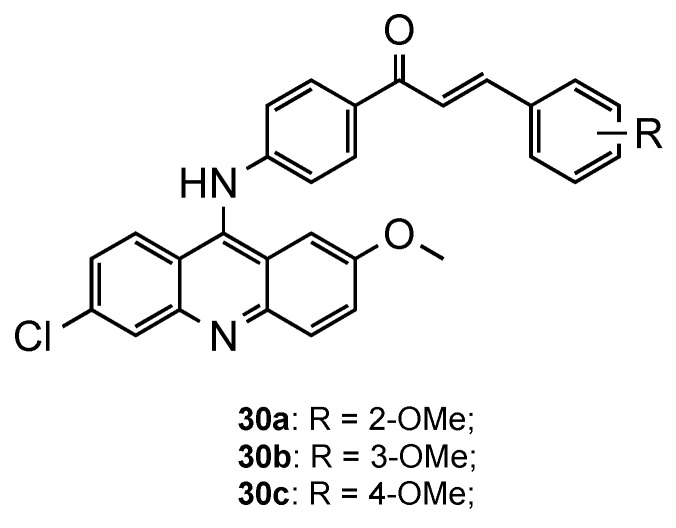
Most promising compounds **30** developed by Prajapati and co-workers [42].

Following the same trend, Prajapati et al. developed another series of acridine–chalcone hybrids [42]. In this case, the authors included the substituents chlorine and methoxy, known to increase antimalarial activity, at positions 6 and 2 of the acridine ring, respectively. Additionally, they also explored the influence of the substituents and respective position on chalcone’s ring B. The in vitro antimalarial activity was next assessed against CQS *Pf* 3D7 and CQR *Pf* Dd2 strains, and the cytotoxicity against the HeLa cell line. Overall, structure–activity results suggested that the monomethoxy substitution significantly increased antimalarial activity, mainly at the 4-position of the ring, when compared to other tested substituents, which supports the results of Tomar and colleagues [41]. Compounds **30a**–**c** (Figure 19) were the most potent, with IC_50_ values in the range of 0.30–0.52 μM and 0.15–0.32 μM against the *Pf* 3D7 and *Pf* Dd2 strains, respectively. Additionally, all the compounds exhibited high selective and resistance indices [42].

### 3.5. Acridine–Cinnamoyl Hybrids

**Figure 20:**
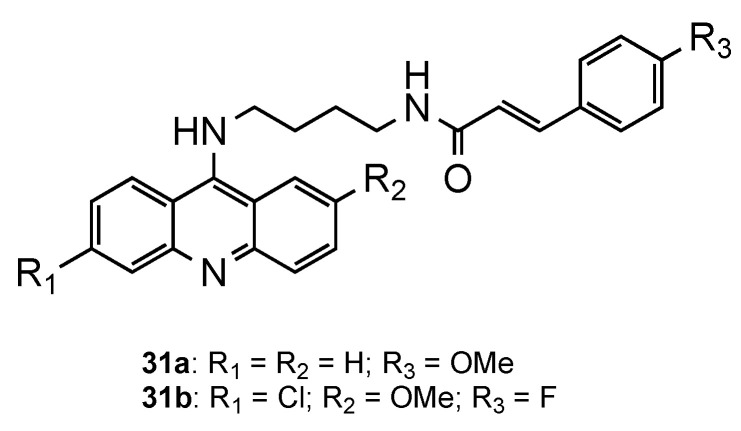
Acridine–cinnamic acid conjugates **31** development by Gomes and co-workers [46,47].

The potential of classical antimalarial drugs conjugated with cinnamoyl moiety was exhaustively explored by Pérez et al. [43,44,45,46,47]. In one of the studies, the authors reported a new class of hybrids combining the acridine core with a cinnamoyl moiety through an aminobutyl chain [46]. The compounds displayed mid-nanomolar in vitro activity against erythrocytic stages of the CQR *Pf* W2 strain (126 nM < IC_50_ < 345 nM). Compound **31a** (Figure 20) showed not only a similar activity in the blood stage (IC_50_ = 138 nM) to **CQ** (IC_50_ = 138 nM), as it also presented activity against hepatic forms of *P. berghei* three times higher (IC_50_ = 3.2 μM) than the reference **PQ** (IC_50_ = 8 μM). At the time, this was an unprecedented result, since **QN** derivatives were described as devoid of activity against *Plasmodium* hepatic forms. Moreover, all the compounds proved to be non-toxic when tested in vitro on Huh7 human hepatoma cells [46].

Later, the same authors [47] synthesized the same hybrids mentioned above, but including the 6-chloro and 2-methoxy substituents on the aminoacridine core (Figure 20). The results of the in vitro antimalarial activity confirmed once more that these substituents significantly improved the antimalarial activity against blood-stage parasites with promising antimalarial activities against CQS *Pf* 3D7 and CQR *Pf* Dd2 strains. The best compound (**31b**, Figure 20) exhibited (i) comparable or better activities than **CQ** against the blood forms of the *Pf* strains 3D7, Dd2 and W2 (**31b:** IC_50_(3D7) = 29.8 nM, IC_50_(Dd2) = 131.0 nM, IC_50_(W2) = 17.8 nM; **CQ:** IC_50_(3D7) = 21.0 nM, IC_50_(Dd2) = 107.5 nM, IC_50_(W2) = 225.8 nM); ii) better activity against the *P. berghei* hepatic stage than **PQ** (**31b:** IC_50_ = 1.6 μM; **PQ:** IC_50_ = 7.5 μM); and iii) low cytotoxicity against human HepG2 cells (SI = 1257) [47].

### 3.6. Other Acridine Hybrids

**Figure 21:**
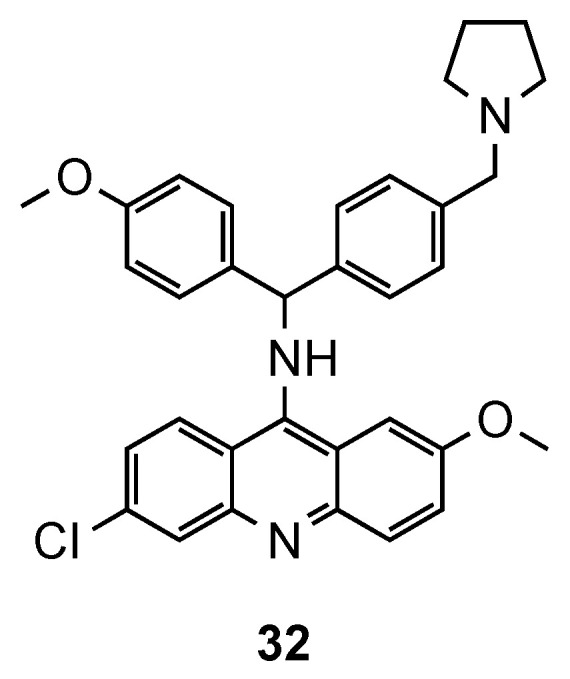
Compound **32** developed by Gemma and co-workers et al. [50].

The synthesis of hybrids containing the acridine and clotrimazole-like pharmacophore was first described by Sandra Gemma et al. [48,49]. In previous works [48,49], the authors changed the chemical structure of clotrimazole, an antimycotic drug, to improve its antimalarial activity. In their more recent work [50], this resulting compound was next linked to 9-aminoacridine and assessed against three *Pf* CQS (D10, 3D7 and NF54) and two CQR (W2 and K1) strains. In the in vitro assays, except for the CQS D10 strain, compound **32** (Figure 21) exhibited better activities (IC_50_ values ranging from 1.0 to 49 nM for CQS D10, 3D7, and NF54 strains and from 9.0 to 59 nM for CQR W2, and K1 strains) than the reference **CQ**. This compound has a polyarylmethyl, which gives it the ability to selectively interact with the free heme-iron center and consequently accumulate into the food vacuole, promoting the generation of toxic radical species for plasmodium’s parasites [50].

**Figure 22:**
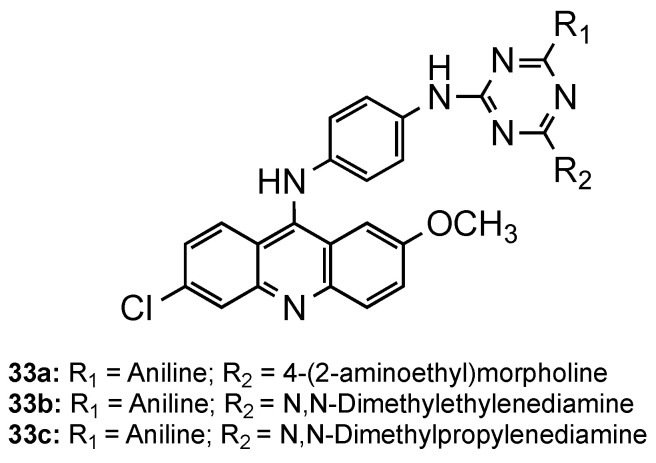
Triazine–acridine hybrids **33** developed by Kumar and co-workers [51].

Kumar et al. directed their attention to triazine–acridine hybrids (**33**, Figure 22) [51]. The synthesized compounds were evaluated for their in vitro antimalarial activity against the CQS *Pf* strain and their cytotoxicity on the VERO cell line. Of the whole set, compounds **33a**–**c** exhibited good antimalarial activities in vitro with a high selectivity index, some of them being better than **CQ** (**33a:** IC_50_ = 6.97 nM, SI = 2896.02; **33b:** IC_50_ = 4.21 nM, SI = 295.02; **33c:** IC_50_ = 4.27 nM, SI = 315.39; **CQ:** IC_50_ = 8.15 nM, SI = 8983). Compound **33a** was further subjected to in vivo study against the CQR N-67 strain of *P. yoelii* orally in Swiss mice at a dose of 100 mg/kg for four days. Although the compound could suppress the infection in 96.59%, it could not provide significant protection to the treated mice in 28 days survival assay [51].

**Figure 23:**
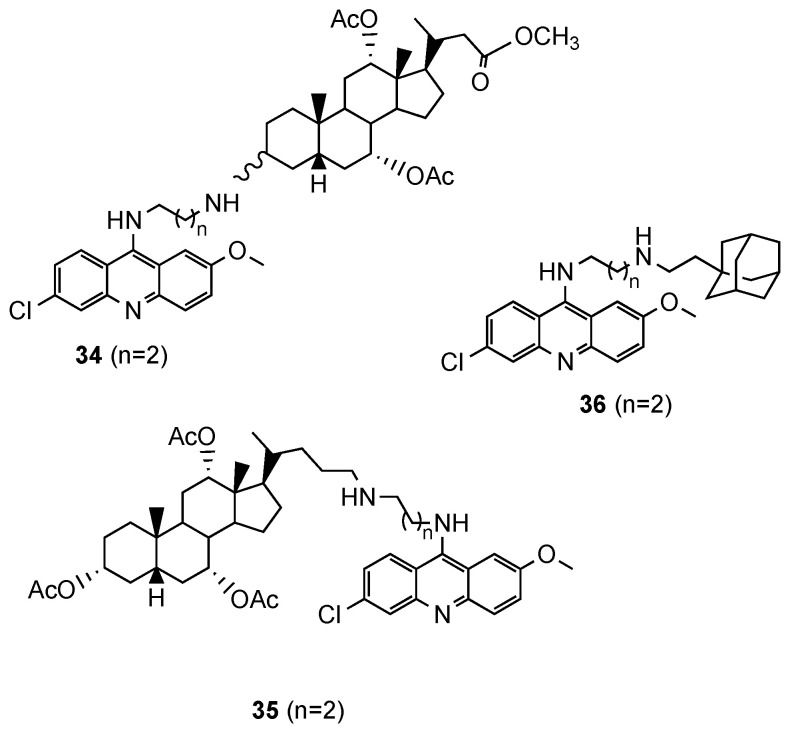
Steroidal (**34** and **35**) and adamantane (**36**) aminoacridine derivatives synthesized by Tot and co-workers [53].

Based on reports concerning the good inhibitory activity of steroidal 4-aminoquinolines, and adamantyl-aminoquinolines [52], Tot et al. [53] expanded their work towards the synthesis and in vitro antimalarial activity of news 9-aminoalkylaminoacridine derivatives possessing steroid and adamantane carriers (**34** and **35,** and **36**, respectively; Figure 23). Compound **36**, an adamantyl derivative, presented an antimalarial activity comparable to that of artemisinin and better than **CQ** against the *Pf* CQS D6, CQR W2 and multi-drug resistant TM91C235 strains (**36:** IC_50_(W2) = 8.6 nM, IC_50_(D6) = 9.3 nM, IC_50_(TM91C235) = 5.8 nM; Artemisinin: IC_50_(W2) = 6.70 nM, IC_50_(D6) = 9.00 nM, IC_50_(TM91C235) = 13.40 nM; **CQ**: IC_50_(W2) = 456.20 nM, IC_50_(D6) = 12.27 nM, IC_50_(TM91C235) = 138.82 nM). Compound cytotoxicity was evaluated in the human liver carcinoma cell line HepG2, and compound **36** exhibited the best selectivity index (SI(W2) = 352; SI(D6) = 326; SI(TM91C235) = 522) [53].

**Figure 24:**
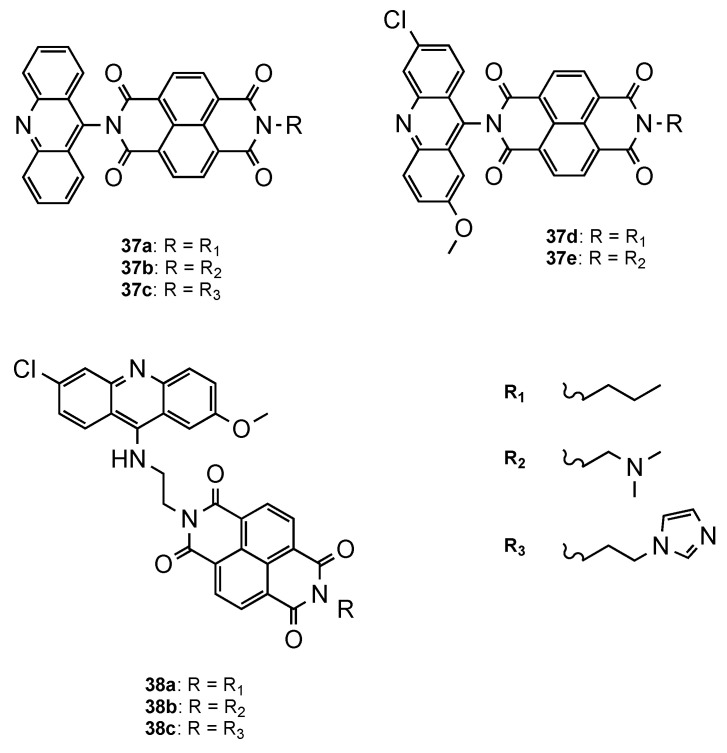
Orthogonal (**37a**–**e**) and flexible (**38a**–**c**) series of NDI–acridine hybrids developed by Dana and co-workers [54].

Another approach was proposed by Dana et al. [54], who developed a novel class of hybrids integrating acridine and redox-active naphthalenediimide (NDI) scaffolds either directly linked (**37a**–**e**, Figure 24) or through a flexible linker (**38a**–**c**, Figure 24). Both series were further evaluated against *Pf* CQS 3D7 and CQR W2 strains. Overall, the compounds showed better activities than the parent drugs NDI and acridine (NDI: IC_50_ = 260 nM; Acridine: IC_50_ = 26.4 nM). The orthogonal series (**37a**–**e**) displayed considerably less antiplasmodial activity than the flexible series (**38a**–**c**), which demonstrated the influence of the spacer linker on the antimalarial activity (**37a**–**e:** 419 < IC_50_ < 4197 nM; **38a**–**c**: 3.65 < IC_50_ < 8.44 nM). Moreover, structure–activity relationship studies proved once again the importance, for antiplasmodial activity, of the incorporation of the substituents 6-chloro and 2-methoxy in the acridine core. Hybrids **38a** and **38b** presented the best activities, alongside no cytotoxic in mammalian fibroblast NIH3T3 cells (**38a**: IC_50_(3D7) = 3.65 nM, IC_50_(W2) = 52.20 nM; **38b**: IC_50_(3D7) = 4.33 nM, IC_50_(W2) = 28.53 nM; **CQ**: IC_50_(3D7) = 12.56 nM, IC_50_(W2) = 430.60 nM) [54].

**Figure 25:**
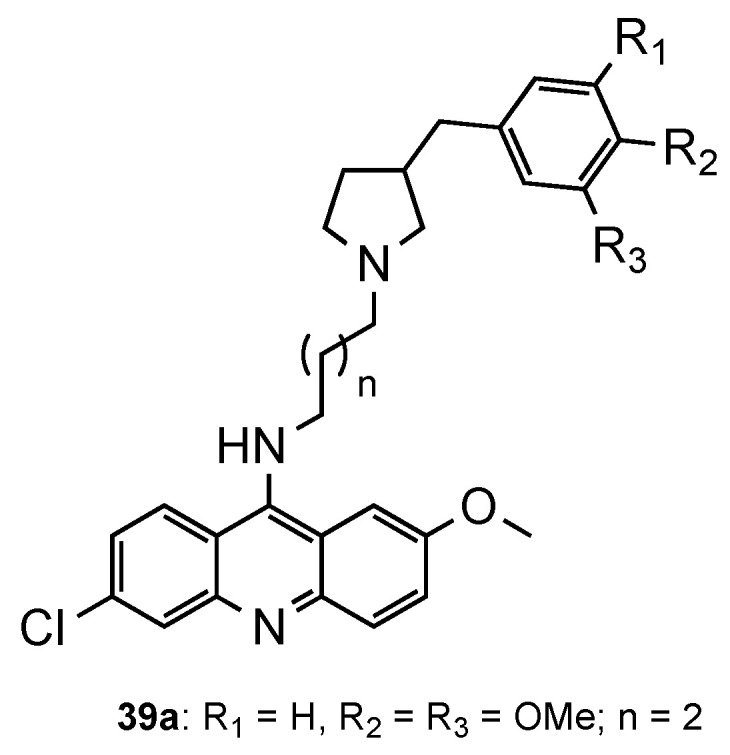
Most active compound **39a** developed by Pandey and co-workers [55].

Considering the recent reports of resistance to ACT [3], Pandey et al. [55] developed a series of pyrrolidinoaminoalkane–acridine hybrids (**39**, Figure 25) to posteriorly use them as potential partners with artemisinin derivatives for the ACT. The two points of structural variations were the phenyl ring and the alkane spacer. When evaluated against the CQS 3D7 and the CQR K1 strains, compound **39a** performed better (**39a**: IC_50_ (3D7) = 9.3 nM, IC_50_ (K1) = 5.2 nM). Then, **39a** was evaluated in vivo in Swiss mice infected with the MDR strain *P. yoelii nigeriensis* at a dose of 100 mg/kg per day for 4 days via the oral route, showing > 90% suppression of parasitaemia on day 4. The authors further showed that, when tested in combination with artemether, arteether or artesunate, compound **39a** serves as a good partner in ACT, namely, with artemether [55].

**Figure 26:**
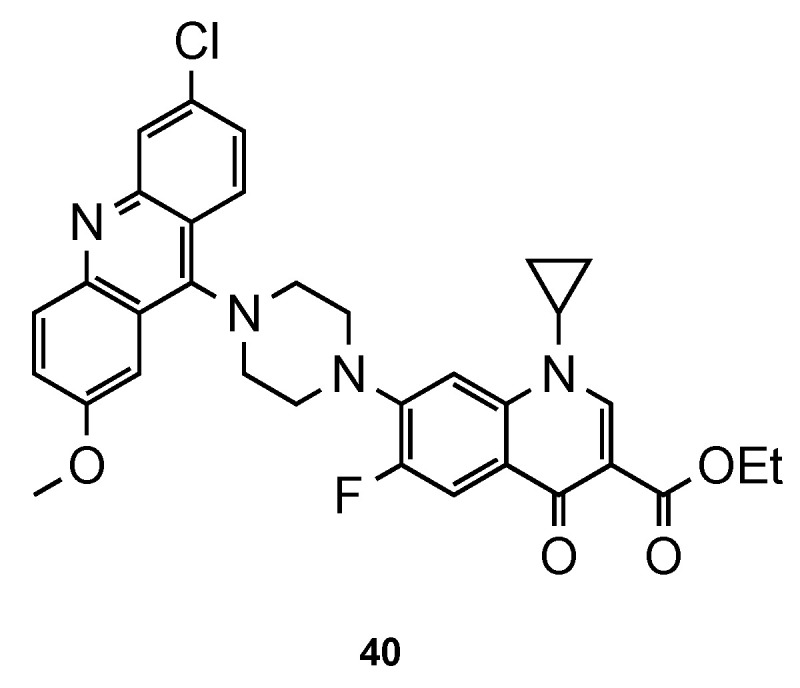
CFX–acridine hybrid **40** developed by Dana and co-workers [56].

Recently, Dana et al. [56] focused their work on ciprofloxacin–acridine hybrids. Ciprofloxacin (CFX) is a second-generation fluoroquinolone-based antibiotic, known to be active against the in vitro culture of *Pf* [57,58]. In this work, the authors functionalized CFX in the *N*-terminal of the piperazine moiety with acridine scaffold. Hybrid **40** (Figure 26) was tested for its in vitro antimalarial activity against the CQS 3D7 strain of *Pf*. Although the activity of **40** was improved in comparison to that of CFX, it was not better than the reference **CQ** (3D7: IC_50_ (**40**) = 359.40 nM, IC_50_ (CFX) = 45350.0 nM, IC_50_ (CQ) = 12.56 nM). However, it is noteworthy that compound **40** did not show any hemolysis of infected RBCs or uninfected RBCs at their highest tested concentrations (10–45 µM), which may suggest minimal toxic effects in vivo [56].

**Figure 27:**
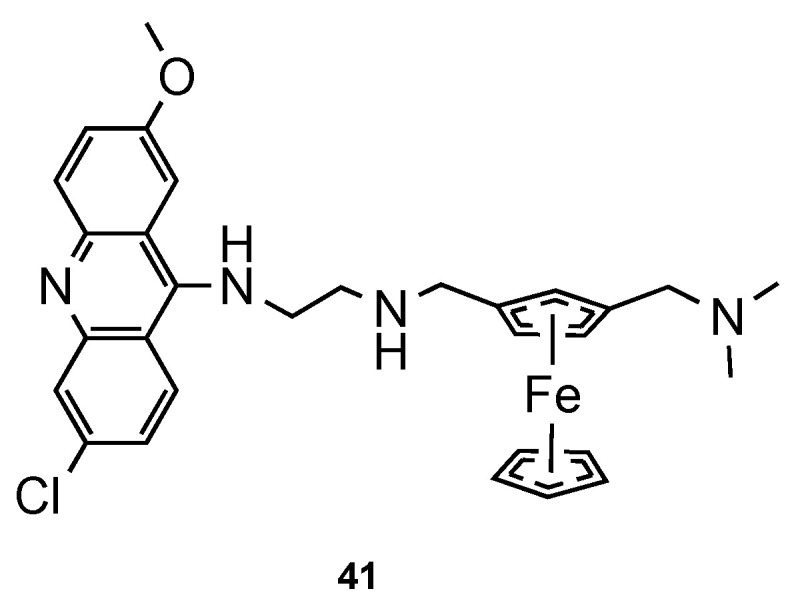
Ferrocene–acridine hybrid **41** developed by Blackie and co-workers [59].

Blackie et al. [59] established the role of ferrocenyl moiety in the antiplasmodial activity of ferroquine, another import scaffold in antimalarial derivatives [60]. The authors concluded that ferrocenyl fragment serves simply as a hydrophobic spacer group. Next, they synthesized compound **41** (Figure 27) and tested them against CQS 3D7 and CQR K1 strains of *Pf*. It is noteworthy that hybrid **41** showed potent antimalarial activity (ED_50_ = 1 and 8 nM against CQS 3D7 and CQR K1 *Pf* strains, respectively), comparatively to **CQ** (ED_50_ = 8.52 and 290 nM against CQS 3D7 and CQR K1 *Pf* strains, respectively) [59].

This review depicts a brief background of the development of antimalarials based on the heteroaromatic core of **QN** focused on the last two decades. Despite the potent antimalarial activity of **QN**, its toxicity was among the reasons for its rapid substitution by **CQ**. Thus, all the modifications described in this review were mainly driven to increase the antimalarial activity of QN derivatives but also to improve their pharmacokinetic and pharmacodynamic properties, as well as overcome problems of cytotoxicity. The classical modifications in the side-chain of acridine reported in this paper emphasized the importance of the substituents methoxy and chlorine at positions 2 and 6 of the acridine ring, respectively. Still, it was also clear that small changes in the chemical structure of the molecules cause major impacts in their antimalarial activity. Alongside these modifications, this review also summarized the advances made towards hybrid molecules holding the acridine moiety. By joining two different molecules with a distinct mechanism of action, it is possible not only to improve the antiplasmodial activity but also to overcome resistance problems, often associated with classical antimalarial compounds. Although this approach does not always fulfill its purpose, it highlights the importance of the nature of the linker between the two moieties. It is noteworthy that the reported efforts have been progressively unveiling novel acridine-based derivatives as promising leads that will hopefully guide future research on the development of acridine-related molecules for the treatment of malaria.

## Sample Availability

Samples of the compounds are not available from the authors.
